# Semaglutide-Induced Small Bowel Pseudo-Obstruction and Ileitis in a Patient With Type 2 Diabetes: A Case Report

**DOI:** 10.7759/cureus.88350

**Published:** 2025-07-20

**Authors:** Faiza Javed, Iman A. A Shaat, Samson O Oyibo

**Affiliations:** 1 Acute Medicine, Peterborough City Hospital, Peterborough, GBR; 2 General Medicine, Peterborough City Hospital, Peterborough, GBR; 3 Diabetes and Endocrinology, Peterborough City Hospital, Peterborough, GBR

**Keywords:** colonic pseudo-obstruction, glucagon-like peptide-1 receptor agonist, ileitis, semaglutide, type 2 diabetes mellites

## Abstract

Type 2 diabetes mellitus is a major health burden globally, with increasing use of glucagon-like peptide-1 receptor agonists (GLP-1RAs) such as semaglutide for glycemic control and cardiovascular risk reduction. While generally well tolerated, GLP-1RAs have been associated with gastrointestinal side effects, including rare reports of bowel obstruction. We describe a case of a 39-year-old male with type 2 diabetes who presented with nausea, vomiting, and abdominal pain five weeks after starting semaglutide. He had four weekly subcutaneous doses of semaglutide 0.25 mg, followed by one dose of 0.5 mg on the fifth week. Examination demonstrated a tender, distended abdomen. Imaging revealed small bowel pseudo-obstruction accompanied by ileitis, with no mechanical cause identified. A diagnosis of bowel pseudo-obstruction was made after exclusion of other causes, including infection, inflammatory bowel disease, and mechanical obstruction. The patient improved with conservative management following drug cessation. Patient education and regular monitoring are crucial, especially for those reporting persistent abdominal symptoms while taking a GLP-1RA for the control of diabetes or weight loss.

## Introduction

Diabetes mellitus is one of the most prevalent health conditions globally. Among those affected, approximately 90% have type 2 diabetes. Alarmingly, it is estimated that around 1 million people in the UK are living with undiagnosed type 2 diabetes, presenting significant public health and clinical challenges related to delayed diagnosis and management [1]. Furthermore, cardiovascular complications secondary to type 2 diabetes mellitus remain a leading cause of morbidity and mortality globally [2].

In recent years, alongside lifestyle interventions and first-line treatment with metformin, glucagon-like peptide-1 receptor agonists (GLP-1RAs) have emerged as a widely used adjunct in antidiabetic therapy [3]. These agents mimic the action of endogenous glucagon-like peptide 1 (GLP-1), which is an incretin hormone released by the gut in response to food intake. GLP-1 suppresses glucagon secretion, stimulates insulin release, slows down gastric emptying, and increases satiety, reducing food intake. The use of a GLP-1RA is indicated in patients with type 2 diabetes as monotherapy (if metformin is inappropriate), or in combination with other drugs (including insulin) if existing treatment fails to achieve adequate glycemic control [3-5].

Although GLP-1RAs have demonstrated efficacy in improving glycaemic control and promoting weight loss, thereby reducing cardiovascular risk, they are also associated with gastrointestinal side effects such as nausea, vomiting, and diarrhoea as well as more serious conditions, including pancreatitis and bowel obstruction. Previous studies have highlighted the potential association between incretin-based therapies and an increased risk of bowel obstruction regardless of diabetes status or not [6,7]. Here, we report the case of a 39-year-old man who developed bowel pseudo-obstruction while taking semaglutide (a GLP-1RA) for the management of type 2 diabetes. This incident was appropriately reported to the Medicines and Healthcare products Regulatory Agency (MHRA), United Kingdom.

## Case presentation

Medical history and demographics

A 39-year-old man presented to the emergency department with an acute onset of nausea and bilious vomiting accompanied by severe, generalised abdominal pain and distension. He did not report any fever, haematemesis, or bleeding per rectum. He admitted that he had been experiencing intermittent mild abdominal discomfort and nausea since starting semaglutide five weeks prior. The semaglutide was started because he was not achieving adequate glycemic control on his existing antidiabetic therapy. He continued to pass stool and flatus as normal until presentation. There was no recent travel or exposure to infectious contacts, and food poisoning was not suspected. There was no family history of any significance.

His medical history included type 2 diabetes, anxiety, and depression. Surgical history included an appendectomy for a perforated appendix 30 years ago. Medications included dapagliflozin 10 mg daily, metformin 1 g twice a day, and the recently started semaglutide 0.5 mg subcutaneous injection once a week for the diabetes, and venlafaxine 150 mg once a day and mirtazapine 45 mg once a day for the anxiety and depression. The semaglutide was started at a dose of 0.25 mg once a week for four weeks, followed by 0.5 mg once a week just before presentation. He had his last dose a few hours before the onset of these severe symptoms. He had no past history of bowel symptoms before starting semaglutide. He was a non-smoker and did not drink alcohol or use recreational drugs.

On examination, he was visibly distressed, and his mucosa was dry. He had a temperature of 36.2°C, a heart rate of 105 beats per minute, a respiratory rate of 16 breaths per minute, a blood pressure of 115/80 mmHg, and an oxygen saturation of 99% on room air. His abdomen was markedly distended with a visible midline scar from a previous laparotomy with generalised abdominal tenderness, most pronounced in the epigastric region. There were no signs of peritonism, no signs of organomegaly, and bowel sounds were present. Digital rectal examination demonstrated an empty rectum. His body mass index was 29 kg/m2.

Investigations

**Figure 1:**
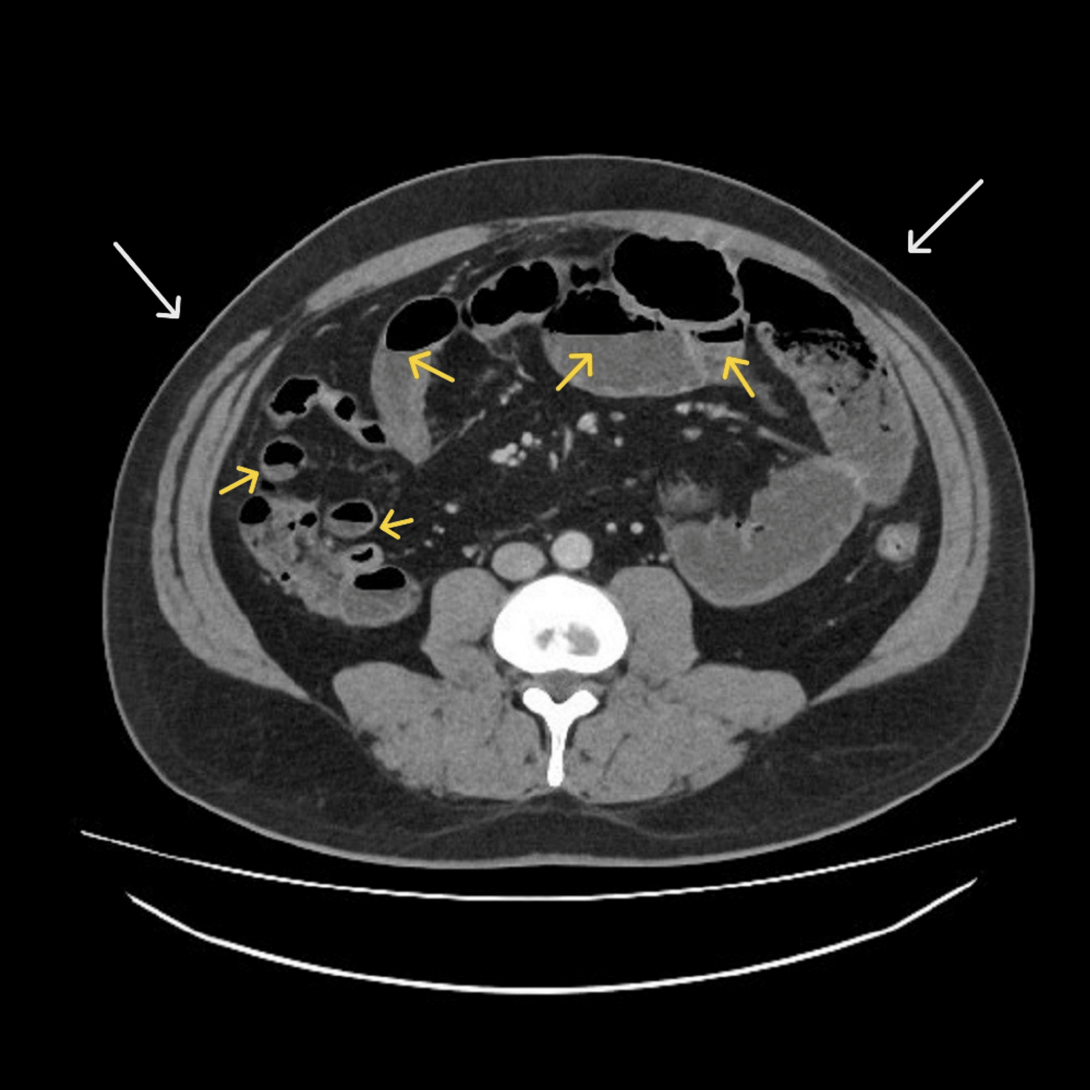
Contrast computerized tomography scan of the abdomen Demonstrating a distended abdomen (white arrows) and dilated bowel loops with multiple air-fluid levels (yellow arrows)

The initial investigations revealed slightly elevated serum C-reactive protein, alanine transferase, and glucose levels (Table 1). His glycated haemoglobin value was elevated, indicating poor diabetes control. A full antibody screen for connective tissue disorders and vasculitis was negative (Table 1). Fecal calprotectin levels were markedly elevated (1188 mg/kg; normal range <50). A contrast computed tomography (CT) scan of the abdomen and pelvis demonstrated multiple dilated proximal small bowel loops (up to 4 cm in diameter) with fecalization and air-fluid levels. There was mild to moderate mural thickening and oedema. A small amount of pelvic fluid was noted, but no free air abscess was seen. A radiological diagnosis of small bowel ileitis and pseudo-obstruction was suggested (Figure 1).

**Table 1:** Initial blood test results

Blood parameters	Results	Reference range
Haemoglobin (g/L)	163	130-180
White cell count (10^9^/L)	10	4-11
Platelet count (10^9^/L)	292	150-400
Sodium (mmol/L)	135	133-146
Potassium (mmol/L)	4.1	3.5-5.3
Chloride (mmol/L)	100	95-108
Creatinine (µmol/L)	82	59-104
Urea (mmol/L)	5.3	2.5-7.8
Glycated haemoglobin (mmol/mol)	84	20-42
C-reactive protein (mg/L)	13	<5
Amylase (IU/L)	50	<100
Lactate (mmol/L)	1.6	0.6-2.5
Total bilirubin (µmol/L)	21	<21
Alkaline phosphatase (U/L)	93	30-130
Alanine transferase (U/L)	48	<41
Albumin (g/L)	46	35-50
Adjusted calcium (mmol/L)	2.2	2.2-2.6
Thyroid stimulating hormone (mU/L)	0.93	0.3-4.2
Random glucose (mmol/L)	9.8	3.9-7.0
Prothrombin time (seconds)	11.7	9.4-16.4
Activated plasma thromboplastin time (seconds)	26	24-36
Anti-nuclear antibodies (ANA)	0.3	<0.7
Neutrophil cytoplasmic antibodies (ANCA)	Negative	Negative

Treatment

Since no other cause was found, a working diagnosis of semaglutide-induced small bowel pseudo-obstruction was made. A nasogastric tube was inserted for suction to decompress the stomach. The patient was kept nil by mouth and treated conservatively with intravenous fluids, regular antiemetics (ondansetron 4 mg and cyclizine 50 mg as required three times a day) and a proton pump inhibitor (intravenous omeprazole 40 mg once a day) for 48 hours.

Outcome and follow-up

The patient's symptoms improved by day three of admission. His abdominal distension had resolved with no more nausea or vomiting. Bowel movements resumed; fecal immunochemical test and stool cultures were negative, ruling out any occult blood in the stool and bowel infection. The nasogastric tube was removed, and the patient was allowed a soft diet. By day four of admission, he was discharged from the hospital. His last dose of the weekly semaglutide injection was just before the admission, so he was instructed not to restart this or take it again in the future. He had no bowel symptoms thereafter.

Because of the elevated fecal calprotectin level during admission, an outpatient gastroscopy and colonoscopy were performed four weeks post-discharge to look for any other causes for bowel inflammation. The gastroscopy demonstrated Barrett’s oesophagus only, and the colonoscopy revealed no evidence of ileitis. Additionally, a repeat fecal calprotectin level was near-normal at 54 mg/kg three weeks after the first test. These findings, combined with the clinical resolution of symptoms, strongly suggest that the initial CT finding of ileitis was transient and related to semaglutide use rather than an underlying chronic inflammatory condition. The patient continued to improve his diet while on his existing antidiabetic medications (dapagliflozin and metformin). Five weeks post-discharge, the patient was feeling fine, and his diabetes control had improved as demonstrated by a repeat glycated haemoglobin value of 58 mmol/mol.

## Discussion

We have reported the case of a man who developed small bowel pseudo-obstruction accompanied by ileitis while taking a GLP-1RA for the management of type 2 diabetes. The dechallenge test was positive in this case (i.e., no return of symptoms after stopping the semaglutide). Hence, as per the World Health Organization-Uppsala Monitoring Center (WHO-UMC) system for standardized case causality assessment, the causal relationship between the administration of semaglutide and the onset of bowel pseudo-obstruction in this case would be regarded as probable or likely [8].

A comprehensive differential diagnosis was considered. He had no past history of bowel symptoms or irritable bowel syndrome (IBS), and showed no features of depression or psychosis during admission. He had been taking metformin for several years without any of the usual metformin-related side-effects (e.g., diarrhoea, nausea, abdominal discomfort, indigestion, flatulence) [9]. Infectious ileitis was ruled out based on negative stool cultures and the absence of systemic signs such as fever or leukocytosis. Inflammatory bowel disease was deemed unlikely given the lack of personal or family history and no clinical or endoscopic evidence of disease activity. Hypothyroidism-induced bowel pseudo-obstruction was ruled out by a normal thyroid hormone result. A mechanical small bowel obstruction was excluded on imaging, which showed no discrete transition point and instead demonstrated features consistent with small bowel pseudo-obstruction, including mural thickening and upstream bowel dilatation. Rectal fecal impaction was ruled out by imaging and digital rectal examination. Elevated fecal calprotectin levels can be due to other inflammatory bowel conditions such as Crohn's disease, ulcerative colitis, infection, polyps, neoplasia, and non-steroidal anti-inflammatory drug-induced enteritis [10]. Subsequent gastroscopy and colonoscopy ruled out any pathological condition, suggesting that the initial elevated calprotectin level was due to the accompanying ileitis. Based on the temporal relationship with the recent use of semaglutide for a five-week duration, along with the exclusion of other plausible causes, a working diagnosis of semaglutide-induced small bowel pseudo-obstruction accompanied by ileitis was established.

Intestinal pseudo-obstruction is characterized by symptoms and signs of bowel obstruction without an obstructive lesion. It can be acute or chronic. Acute cases can be due to trauma, drugs, and infections, while chronic cases can be due to systemic diseases, amyloidosis, scleroderma, and neurological disorders [11]. It is postulated that there is an imbalance between the excitatory and inhibitory functions within the intestinal neuromuscular bundle, resulting in motor impairment and bowel dilatation [11]. As for GLP-1RAs, they slow down intestinal motility and transit time, increasing the risk of food and fluid build-up and bowel obstruction [12].

This case raises important concerns about a possible gastrointestinal adverse effect associated with the use of GLP-1RAs. Although rare, pseudo-obstruction can mimic mechanical obstruction with symptoms such as abdominal pain, distension, and altered bowel habits despite the absence of a mechanical lesion. Bowel obstruction related to GLP-1RA has been documented in a few case reports. For instance, one case report described a 29-year-old man who developed gastric outlet obstruction while taking semaglutide. He recovered after three days of conservative management [13]. Two other published case reports described patients who developed bowel obstruction due to intussusception while on liraglutide and semaglutide, respectively, suggesting a potential link between these agents and abnormal bowel movement or structural changes in the gut [14,15]. However, these remain rare events, and causality is difficult to establish due to confounding factors such as co-existing gastrointestinal conditions, previous surgeries, or underlying inflammatory bowel disease.

The finding of ileitis in our patient further complicated the picture. Inflammation of the ileum, potentially exacerbated by altered gut motility or local mucosal irritation, could have led to a state of functional obstruction or pseudo-obstruction. Whether the ileitis was directly due to the semaglutide and then became a predisposing factor towards pseudo-obstruction or the ileitis was due to the obstructive pressure on the bowel mucosa is unclear. Nevertheless, clinicians should maintain a high index of suspicion when patients on GLP-1RA therapy present with new or worsening gastrointestinal symptoms.

## Conclusions

This case emphasizes the need for further pharmacovigilance and clinical research into the gastrointestinal safety profile of the GLP-1RA class of drugs. Prospective studies or registry data could help elucidate the frequency and mechanisms behind these rare but serious adverse events. When trying to establish causality it is important to record the onset and relief of symptoms in relation to the offending drug, and to rule out psychotic conditions. In the meantime, patient education and regular monitoring are crucial, especially for those reporting persistent abdominal symptoms while taking a GLP-1RA for the control of diabetes or weight loss.
